# Supplementary material for: A New Method to Monitor the Nutritional Quality of Packaged Foods in the Global Food Supply in Order to Provide Feasible Targets for Reformulation
Source: Nutrients. 2021 Feb 9;13(2):576. doi: 10.3390/nu13020576 (PMC7916088; doi:10.3390/nu13020576)
Supplement: Supplementary file 1 [file nutrients-13-00576-s001.pdf]

Supplementary material

Table 1. Initial Categorization of products (A), number of considered products and number of products kept for analyses (B), category relevant nutrients (C) and final number of categories (D).

**A. Initial Categorization**					**B. Products**		**C. Category relevant Nutrients**					**D. Split**
**MINTEL.Category**	**MINTEL.Sub.Category**	**Liq.Sol**	**Dilution**	**INITIAL.Category**	**N-initial**	**N-Kept**	**saturated fat**	**total sugars**	**total sodium**	**protein**	**fiber**	**FINAL.Categories**
TOTAL					442,018	350,994						263
Bakery	Baking Ingredients & Mixes	Sol	1	Baking Ingredients & Mixes	10,928	10,545	no	no	no	yes	no	1
Bakery	Bread & Bread Products	Sol	1	Bread & Bread Products	10,749	10,106	no	no	yes	yes	no	1
Bakery	Cakes, Pastries & Sweet Goods	Sol	1	Cakes, Pastries & Sweet Goods	15,231	11,291	yes	yes	yes	yes	no	1
Bakery	Savoury Biscuits Crackers	Sol	1	Savoury Biscuits Crackers	6308	5254	yes	no	yes	yes	no	4
Bakery	Sweet Biscuits Cookies	Sol	1	Sweet Biscuits Cookies	25,439	18,316	yes	yes	yes	yes	no	4
Breakfast Cereals	Cold Cereals	Sol	1	Cold Cereals	9375	7673	no	yes	yes	yes	yes	24
Breakfast Cereals	Hot Cereals	Liq	1	Hot Cereals	2761	2019	yes	no	no	yes	yes	1
Carbonated Soft Drinks	Carbonated Soft Drinks	Liq	1	Carbonated Soft Drinks	8279	7122	no	yes	no	no	no	2
Chocolate Confectionery	Seasonal Chocolate	Sol	1	Chocolate Confectionery	27397	23,403	yes	yes	no	yes	no	2
Dairy	Butter	Sol	1	Butter	1758	1425	yes	no	no	no	no	1
Dairy	Cream	Sol	1	Cream	1375	1159	yes	no	no	no	no	1
Dairy	Creamers	Liq	1	Creamers Liquid or Reconstituted	272	238	yes	yes	no	no	no	4
Dairy	Creamers	Liq	1	Creamers Powder	246	150	yes	yes	no	no	no	3
Dairy	Curd & Quark	Sol	1	Curd & Quark	1014	738	yes	no	no	yes	no	1
Dairy	Drinking Yogurt & Liquid Cultured Milk	Liq	1	Drinking Yogurt & Liquid Cultured Milk Liquid	5444	3121	no	yes	no	yes	no	1

Dairy	Evaporated Milk	Liq	1	Evaporated Milk Liquid	143	115	yes	yes	no	yes	no	1
Dairy	Flavoured Milk	Liq	1	Flavoured Milk Liquid	2885	1883	yes	yes	no	yes	no	1
Dairy	Fresh Cheese & Cream Cheese	Sol	1	Fresh Cheese & Cream Cheese	1880	1710	yes	no	yes	yes	no	2
Dairy	Hard Cheese & Semi-Hard Cheese	Sol	1	Hard Cheese & Semi-Hard Cheese	7126	6532	yes	no	yes	yes	no	1
Dairy	Margarine & Other Blends	Sol	1	Margarine & Other Blends	968	850	yes	no	yes	no	no	3
Dairy	Plant Based Drinks (Dairy Alternatives)	Liq	1	Plant Based Drinks (Dairy Alternatives) Liquid	3333	2885	no	yes	no	no	no	2
Dairy	Plant Based Spoonable Yogurts (Dairy Alternatives)	Sol	1	Plant Based Spoonable Yogurts (Dairy Alternatives)	723	676	no	yes	no	no	no	1
Dairy	Processed Cheese	Sol	1	Processed Cheese	2795	2346	yes	no	yes	yes	no	1
Dairy	Soft Cheese & Semi-Soft Cheese	Sol	1	Soft Cheese & Semi-Soft Cheese	5058	4577	yes	no	yes	yes	no	2
Dairy	Soft Cheese Desserts	Sol	1	Soft Cheese Desserts	936	758	yes	yes	no	yes	no	1
Dairy	Spoonable Yogurt	Sol	1	Spoonable Yogurt	8301	6569	yes	yes	no	no	no	2
Dairy	Sweetened Condensed Milk	Liq	1	Sweetened Condensed Milk Liquid	315	182	yes	yes	no	yes	no	1
Dairy	White Milk	Liq	1	White Milk Liquid	3505	2248	yes	yes	no	yes	no	1
Desserts & Ice Cream	Chilled Desserts	Sol	1	Chilled Desserts	3940	3282	yes	yes	no	no	no	1
Desserts & Ice Cream	Dairy Based Ice Cream & Frozen Yogurt	Sol	1	Dairy Based Ice Cream & Frozen Yogurt G	2571	2208	yes	yes	no	no	no	4
Desserts & Ice Cream	Dairy Based Ice Cream & Frozen Yogurt	Sol	1	Dairy Based Ice Cream & Frozen Yogurt G Sandwich	151	140	yes	yes	yes	no	no	1

Desserts & Ice Cream	Dairy Based Ice Cream & Frozen Yogurt	Sol	1	Dairy Based Ice Cream & Frozen Yogurt ML	2030	1199	yes	yes	no	no	no	4
Desserts & Ice Cream	Dairy Based Ice Cream & Frozen Yogurt	Sol	1	Dairy Based Ice Cream & Frozen Yogurt ML Sandwich	137	0	no	no	no	no	no	0
Desserts & Ice Cream	Dessert Toppings	Sol	1	Dessert Toppings	502	435	no	yes	no	no	no	1
Desserts & Ice Cream	Frozen Desserts	Sol	1	Frozen Desserts	1194	968	yes	yes	no	no	no	1
Desserts & Ice Cream	Plant Based Ice Cream & Frozen Yogurt (Dairy Alternatives)	Sol	1	Plant Based Ice Cream & Frozen Yogurt (Dairy Alternatives)	473	454	yes	yes	no	no	no	1
Desserts & Ice Cream	Shelf-Stable Desserts	Sol	1	Shelf-Stable Desserts	3840	2941	no	yes	no	no	no	1
Desserts & Ice Cream	Water Based Ice Lollies, Pops & Sorbets	Sol	1	Water Based Ice Lollies, Pops & Sorbets	1183	909	no	yes	no	no	no	1
Juice Drinks	Fruit Flavoured Still Drinks	Liq	1	Fruit Flavoured Still Drinks	6175	4748	no	yes	no	no	no	2
Juice Drinks	Nectars	Liq	1	Nectars	4608	3692	no	yes	no	no	no	1
Meals & Meal Centers	Instant Noodles	Sol	1	Instant Noodles	4524	3792	no	no	yes	yes	no	4
Meals & Meal Centers	Instant Pasta	Sol	1	Instant Pasta	392	364	no	no	yes	yes	no	1
Meals & Meal Centers	Instant Rice	Sol	1	Instant Rice	376	302	no	no	yes	yes	no	1
Meals & Meal Centers	Meal Kits	Sol	1	Meal Kits	2199	1989	no	no	yes	yes	no	1
Meals & Meal Centers	Pastry Dishes	Sol	1	Pastry Dishes	1545	1467	yes	no	yes	yes	no	2
Meals & Meal Centers	Pizzas	Sol	1	Pizzas	3454	3320	yes	no	yes	yes	no	1

Meals & Meal Centers	Prepared Meals	Sol	1	Prepared Meals	9058	8181	no	no	yes	yes	no	1
Meals & Meal Centers	Salads	Sol	1	Salads	2075	2033	no	no	yes	no	no	1
Meals & Meal Centers	Sandwiches Wraps	Sol	1	Sandwiches Wraps	2108	1944	yes	no	yes	yes	no	1
Other Beverages	Beverage Concentrates	Liq	1	Beverage Concentrates	2189	1798	no	yes	no	no	no	2
Other Beverages	Beverage Mixes	Liq	1	Powdered Beverages Reconstituted	3574	0	no	no	no	no	no	0
Other Beverages	Beverage Mixes	Liq	10	Powdered Beverages wo reconstitution	6939	4819	no	yes	no	no	no	2
Other Beverages	Meal Replacements & Other Drinks	Liq	1	Meal Replacements & Other Drinks Liquid	1857	1435	no	yes	no	yes	no	4
Other Beverages	Meal Replacements & Other Drinks	Liq	10	Meal Replacements & Other Drinks Powder	2985	2762	no	no	no	yes	no	1
Processed Fish, Meat & Egg Products	Eggs & Egg Products	Sol	1	Eggs & Egg Products	2045	1422	yes	no	yes	yes	no	1
Processed Fish, Meat & Egg Products	Fish Products	Sol	1	Fish Products	12,956	11,651	no	no	yes	yes	no	2
Processed Fish, Meat & Egg Products	Meat Products	Sol	1	Meat Products	16,757	14,567	yes	no	yes	yes	no	4
Processed Fish, Meat & Egg Products	Meat Substitutes	Sol	1	Meat Substitutes	3024	2064	no	no	yes	yes	yes	4
Processed Fish, Meat & Egg Products	Poultry Products	Sol	1	Poultry Products	8608	7772	no	no	yes	yes	no	2
RTDs	RTD (Iced) Coffee	Liq	1	RTD (Iced) Coffee	2535	1819	no	yes	no	no	no	2
RTDs	RTD (Iced) Tea	Liq	1	RTD (Iced) Tea	4064	3070	no	yes	no	no	no	2

Sauces & Seasonings	Cooking Sauces	Liq	1	Cooking Sauces na	5313	4200	no	yes	yes	no	no	4
Sauces & Seasonings	Dressings & Vinegar	Sol	1	Dressings & Vinegar	3161	2415	no	yes	yes	no	no	4
Sauces & Seasonings	Mayonnaise	Sol	1	Mayonnaise	1303	1133	yes	no	yes	no	no	1
Sauces & Seasonings	Other Sauces & Seasonings	Sol	1	Other Sauces & Seasonings	1120	865	no	no	yes	yes	no	6
Sauces & Seasonings	Pasta Sauces	Sol	1	Pasta Sauces	3744	3404	no	no	yes	no	no	1
Sauces & Seasonings	Pickled Condiments	Sol	1	Pickled Condiments	5636	5175	no	no	yes	no	no	1
Sauces & Seasonings	Seasonings	Sol	1	Seasonings	7634	5806	no	no	yes	yes	no	4
Sauces & Seasonings	Stocks	Liq	1	Stocks Cubed Reconstituted	147	146	no	no	yes	no	no	2
Sauces & Seasonings	Stocks	Liq	10	Stocks Cubed wo reconstitution	345	302	no	no	yes	no	no	1
Sauces & Seasonings	Stocks	Liq	10	Stocks Granules	384	359	no	no	yes	no	no	2
Sauces & Seasonings	Stocks	Liq	1	Stocks Liquid	531	490	no	no	yes	no	no	2
Sauces & Seasonings	Stocks	Liq	10	Stocks Other	104	101	no	no	yes	no	no	2
Sauces & Seasonings	Table Sauces	Sol	1	Table Sauces	7561	5425	no	yes	yes	no	no	2
Savoury Spreads	Dips	Sol	1	Dips	2030	1855	yes	no	yes	no	no	1
Savoury Spreads	Meat Pastes & Pates	Sol	1	Meat Pastes & Pates	1343	1259	yes	no	yes	yes	no	2
Savoury Spreads	Sandwich Fillers Spreads	Sol	1	Sandwich Fillers Spreads	604	552	yes	no	yes	yes	no	2
Savoury Spreads	Savoury Vegetable Pastes Spreads	Sol	1	Savoury Vegetable Pastes Spreads	1301	1193	yes	no	yes	no	no	1
Side Dishes	Noodles	Sol	1	Noodles	1640	1616	no	no	no	yes	no	2
Side Dishes	Pasta	Sol	1	Pasta	11,119	11,060	no	no	no	yes	no	1

Side Dishes	Potato Products	Sol	1	Potato Products	2741	2606	no	no	yes	no	no	2
Side Dishes	Stuffing, Polenta & Other Side Dishes	Sol	1	Stuffing, Polenta & Other Side Dishes	3671	2569	no	no	no	yes	yes	1
Snacks	Bean-Based Snacks	Sol	1	Bean-Based Snacks	1291	716	no	no	yes	yes	yes	4
Snacks	Cassava & Other Root-Based Snacks	Sol	1	Cassava & Other Root-Based Snacks	1206	874	yes	no	yes	no	no	4
Snacks	Corn-Based Snacks	Sol	1	Corn-Based Snacks	4201	3540	yes	no	yes	yes	no	1
Snacks	Fruit Snacks	Sol	1	Fruit Snacks	7997	4451	no	yes	no	no	yes	1
Snacks	Hors d'oeuvres Canapes	Sol	1	Hors d'oeuvres Canapes	4539	3417	yes	no	yes	yes	no	2
Snacks	Meat Snacks	Sol	1	Meat Snacks	3846	3701	no	no	yes	yes	no	2
Snacks	Nuts	Sol	1	Nuts	8885	4611	yes	no	yes	yes	yes	18
Snacks	Other Snacks	Sol	1	Other Snacks	327	226	yes	no	yes	yes	no	6
Snacks	Popcorn	Sol	1	Popcorn	2336	1628	yes	no	yes	yes	yes	12
Snacks	Potato Snacks	Sol	1	Potato Snacks	8278	6471	yes	no	yes	yes	no	1
Snacks	Rice Snacks	Sol	1	Rice Snacks	1855	1622	no	no	yes	yes	no	2
Snacks	Snack Cereal Energy Bars	Sol	1	Snack Cereal Energy Bars	9016	7125	yes	yes	yes	yes	yes	12
Snacks	Snack Mixes	Sol	1	Snack Mixes	3325	2098	yes	yes	no	yes	yes	4
Snacks	Vegetable Snacks	Sol	1	Vegetable Snacks	2315	1290	no	no	yes	yes	yes	1
Snacks	Wheat & Other Grain-Based Snacks	Sol	1	Wheat & Other Grain-Based Snacks	3929	2880	yes	no	yes	yes	no	2
Soup	Dry Soup	Liq	1	Dry Soups Reconstituted	589	581	no	no	yes	no	no	1
Soup	Dry Soup	Liq	10	Dry Soups wo reconstitution	1371	1085	no	no	yes	no	no	2
Soup	Wet Soup	Liq	1	Wet Soup	3778	3579	no	no	yes	no	no	1
Sugar & Gum Confectionery	Boiled Sweets	Sol	1	Boiled Sweets	1309	899	no	yes	no	no	no	1
Sugar & Gum Confectionery	Gum	Sol	1	Gum	1195	0	no	no	no	no	no	0
Sugar & Gum Confectionery	Liquorice	Sol	1	Liquorice	481	463	no	yes	no	no	no	1

Sugar & Gum Confectionery	Lollipops	Sol	1	Lollipops	535	419	no	yes	no	no	no	1
Sugar & Gum Confectionery	Marshmallows	Sol	1	Marshmallows	740	615	no	yes	no	no	no	1
Sugar & Gum Confectionery	Medicated Confectionery	Sol	1	Medicated Confectionery	688	0	no	no	no	no	no	0
Sugar & Gum Confectionery	Mixed Assortments	Sol	1	Mixed Assortments	111	110	no	yes	no	no	no	1
Sugar & Gum Confectionery	Other Sugar Confectionery	Sol	1	Other Sugar Confectionery	1934	1289	no	yes	no	no	no	1
Sugar & Gum Confectionery	Pastilles, Gums, Jellies & Chews	Sol	1	Pastilles, Gums, Jellies & Chews	5999	4933	no	yes	no	no	no	1
Sugar & Gum Confectionery	Standard & Power Mints	Sol	1	Standard & Power Mints	804	0	no	no	no	no	no	0
Sugar & Gum Confectionery	Sticks, Liquids & Sprays	Liq	1	Sticks, Liquids & Sprays	112	108	no	yes	no	no	no	1
Sugar & Gum Confectionery	Toffees, Caramels & Nougat	Sol	1	Toffees, Caramels & Nougat	2325	1638	yes	yes	no	yes	no	1
Sweet Spreads	Caramel & Cream Spreads	Sol	1	Caramel & Cream Spreads	613	389	yes	yes	yes	yes	no	2
Sweet Spreads	Chocolate Spreads	Sol	1	Chocolate Spreads	1474	1248	yes	yes	no	yes	no	1
Sweet Spreads	Confiture & Fruit Spreads	Sol	1	Confiture & Fruit Spreads	4044	3401	no	yes	no	no	no	1
Sweet Spreads	Nut Spreads	Sol	1	Nut Spreads	1553	1033	yes	yes	no	yes	yes	2
Sweet Spreads	Syrups	Sol	1	Syrups	639	585	no	yes	no	no	no	1
Water	Flavoured Water	Liq	1	Flavoured Water	2334	0	no	no	no	no	no	0
